# The effect of altitude on erythropoiesis-stimulating agent dose, hemoglobin level, and mortality in hemodialysis patients

**DOI:** 10.1007/s40620-016-0350-1

**Published:** 2016-09-19

**Authors:** Scott Sibbel, Bradley J. Maroni, Steven M. Brunelli

**Affiliations:** 1 DaVita Clinical Research, 825 South 8th Street, Minneapolis, MN USA; 2 Akebia Therapeutics, Cambridge, MA USA

**Keywords:** Erythropoietin, Anemia, Hemodialysis, Altitude

## Abstract

Residence at higher altitude has been associated with improved anemia parameters and lower mortality rates among end-stage renal disease (ESRD) patients. However, these associations were observed prior to the 2011 shift in erythropoiesis-stimulating agent (ESA) dosing. To determine the impact of altitude on contemporary ESRD patients, a retrospective observational analysis was conducted in which patients were ascribed to one of four altitude categories as of 1 Jan 2012 and outcomes were assessed during 2012. Associations between altitude category and outcomes were estimated using generalized linear mixed models, adjusted for covariates that differed at baseline. Patients at higher altitude were less likely to receive ESA treatment, and dose was 723 U/treatment (95 % confidence interval [CI]: 544, 834) lower in the highest altitude category compared to the lowest category. The proportion of patients using IV iron decreased with increasing altitude category. Patients in the highest two categories had greater mean hemoglobin values (+0.15 and +0.23 g/dL) than the lowest. Mortality was lower for patients in the highest altitude category compared to those in the lowest (incidence rate ratio 0.73; 95 % CI: 0.63, 0.88), although their rate of missed dialysis treatments was slightly higher. This study confirms that, in the context of current anemia management practices, high altitude is associated with higher hemoglobin and lower mortality, despite lower utilization of ESA and IV iron.

**Electronic supplementary material:**

The online version of this article (doi:10.1007/s40620-016-0350-1) contains supplementary material, which is available to authorized users.

## Introduction

Anemia is a common feature of chronic kidney disease (CKD). It is caused by diminished erythropoietin production within the failing kidney, compounded by other factors, including nutritional deficiencies, inflammation, and blood loss ([1, 2] and references therein). Because anemia is associated with increased morbidity and transfusion risk in patients on dialysis, treatments such as IV iron and erythropoiesis-stimulating agents (ESA) have been fundamental aspects of CKD management for the past two decades [1, 3]. However, treatment with high doses of ESA has been associated with poor survival [4–8].

Erythropoiesis and iron metabolism are responsive to oxygen partial pressure ([9] and references therein). Because oxygen partial pressure varies by altitude, comparison of outcomes among CKD patients residing at or near sea level versus higher altitudes may contribute to an understanding of the factors underlying the risk of adverse outcomes associated with high-dose ESA use. Prior studies that evaluated associations between altitude and outcomes among CKD patients found that those residing at higher altitudes achieved similar or higher hemoglobin (Hb) levels while utilizing less ESA [10, 11], and had reduced mortality [12–14], as compared to those residing closer to sea level. These data support the premise that the deleterious effects of ESA on survival are independent of achieved Hb levels, although the relative contribution of Hb excursions remains unclear.

Importantly, all prior investigations of the impact of altitude on anemia in CKD patients utilized patient data collected prior to 2011. As a result of changes to the ESA labels and reimbursement policy in the United States [15–17], there has been a dramatic decline in the mean ESA dose among users [18, 19] and the percentage of patients receiving very high ESA doses [20] since that time. In this study, we sought to determine whether the associations between altitude, ESA use, and outcomes observed prior to the policy changes of 2011 could be confirmed within the context of contemporary ESA prescribing practices. In particular, we examined anemia management parameters, rates of missed hemodialysis sessions (a surrogate for hospitalizations), and mortality.

## Methods

### Data source and study population

We conducted a retrospective, observational analysis of patients receiving in-center hemodialysis at a large dialysis organization (LDO) in the United States during the period 01 January—31 December 2012. Patients included in the analysis were those who, as of 01 January 2012, were at least 18 years old, were not US Veterans Affairs beneficiaries (contractual stipulation), and had a dialysis vintage of at least 6 months (to allow for equilibration of anemia management after dialysis initiation). Patients were followed forward from 01 January 2012 for 12 months or until censoring due to transfer of care away from the LDO, change in dialysis modality, transplant, recovery of renal function, withdrawal from dialysis, or death. All data were derived from the electronic health records (EHR) of the LDO, which contains information on patient demographics, disease history, comorbidities, dialysis-specific information for each treatment session, laboratory results such as hemoglobin, and IV anemia medications administered at dialysis sessions (ESAs and iron). The LDO uses a single ESA (epoetin alfa), which is delivered intravenously.

This observational study was conducted retrospectively using de-identified patient data; the protocol was reviewed by an institutional review board (Quorum, Seattle, WA) and determined to be exempt. We adhered to the Declaration of Helsinki and informed consent was not required.

### Exposure

The exposure of interest was altitude, which was determined by clinic zip code as of 01 January 2012. Four categories of altitude (1500-foot increments) were used for associative analyses.

### Outcomes

ESA utilization was calculated as the mean per treatment dose (expressed as U/treatment) during the month among all patients, including both ESA users and non-users. ESA use was assessed as the proportion of patients using ESA during the corresponding month. ESA dose among users was calculated as the mean per-treatment dose (expressed as U/treatment) during the month among patients who used ESA. IV iron utilization, use and dose were assessed similarly to ESA except that values were expressed as monthly cumulative values (as opposed to per-treatment values) to accommodate weekly dosing frequency. Hb level was considered as the initial value recorded for each patient during each month of follow-up.

The above data were used to calculate metrics of ESA response. ESA hyporesponsiveness was defined as two consecutive bimonthly hemoglobin measures <10 g/dL in the setting of a concurrent averaged ESA dose >7700 U/treatment in every month. ESA Response Index (ERI) was defined as the mean monthly ESA dose divided by the mean monthly hemoglobin measure in every month.

Mortality was identified through death records and the mortality rate was calculated as number of events divided by the time at risk. Missed dialysis treatment rate was defined as the number of missed dialysis sessions (not rescheduled, identified from LDO records) divided by time at risk.

### Statistical analysis

Baseline patient characteristics were described as means, standard deviations, medians, interquartile ranges, counts, and proportions as dictated by data type. These were compared across altitude categories by ANOVA, Kruskal Wallis tests, and chi squared tests as appropriate.

Continuous outcomes, including ESA utilization and IV iron utilization, were described as means, standard deviations, medians, and interquartile ranges. Differences in use and dose among users of ESA and IV iron were compared using generalized linear mixed models (GLMM) with fixed effects terms for month of follow up and altitude category and with random patient intercepts. Resultant comparisons across altitude categories are expressed as mean differences (95 % confidence interval [CI]). Event outcomes were summarized as counts and proportions. Prevalence across altitude categories was compared using GLMM as above, and expressed as mean differences in prevalence (95 % CI). Rate outcomes were fit assuming a GLMM-type model. The outcome was specified as a negative binomial. Confidence intervals were determined by generating bootstrap estimates with 100 replicates, using unrestricted sampling (IV iron use and ESA use) or robust variance estimators (all other analyses).

All models were adjusted by the addition of covariate terms for patient characteristics that differed significantly across altitude categories at baseline (*P* < 0.10), which were: age, race, etiology, weight, vintage, vascular access type, history of diabetes, congestive heart failure, cerebrovascular disease, alcohol abuse, peripheral vascular disease, and Charlson comorbidity index.

All analyses were performed using SAS, version 9.2 (SAS Institute, Inc).

## Results

Patients were assigned to one of four altitude categories, defined and expressed in terms of feet above sea level, based on the zip code of their clinic: 0–1499 (n = 92,490), 1500–2999 (n = 3118), 3000–4499 (n = 1659), and ≥ 4500 (n = 2027); categories correspond to 0–456.9, 457–914.1, 914.2–1371.3 and ≥1371.4 meters above sea level, respectively. Baseline characteristics of patients in the four altitude categories are shown in Table 1. Compared to patients in the lowest altitude category, patients in the highest altitude category were older with lower dry weight, longer dialysis vintage, and higher Kt/V; were more frequently diabetic and less likely to have a history of hypertension or congestive heart failure; and had lower ferritin values.

**Table 1:** Baseline patient characteristics by altitude

	Altitude category (feet)				
	0–1499 (n = 92,490)	1500–2999 (n = 3118)	3000–4499 (n = 1659)	≥4500 (n = 2027)	*P*-value
Age, years, mean ± SD	61.6 ± 14.9	61.9 ± 14.6	62.3 ± 14.0	63.0 ± 14.8	<0.001
Sex, n (%)					0.41
Male	50,441 (54.5 %)	1657 (53.1 %)	918 (55.3 %)	1099 (54.2 %)	
Female	42,047 (45.5 %)	1461 (46.9 %)	741 (44.7 %)	928 (45.8 %)	
Race, n (%)					<0.001
White	31,185 (33.7 %)	1540 (49.4 %)	359 (21.6 %)	821 (40.5 %)	
Black	38,433 (41.6 %)	446 (14.3 %)	69 (4.2 %)	245 (12.1 %)	
Hispanic	16,007 (17.3 %)	609 (19.5 %)	1146 (69.1 %)	390 (19.2 %)	
Asian	3491 (3.8 %)	137 (4.4 %)	16 (1.0 %)	45 (2.2 %)	
Other/unknown	3374 (3.7 %)	386 (12.4 %)	69 (4.2 %)	526 (26.0 %)	
Etiology ESRD, n (%)					<0.001
Diabetes	41,288 (44.5 %)	1576 (50.6 %)	992 (59.8 %)	1096 (54.1 %)	
Hypertension	29,462 (31.9 %)	785 (25.2 %)	349 (21.0 %)	308 (15.1 %)	
Other/unknown	21,740 (23.5 %)	757 (24.3 %)	318 (19.2 %)	623 (30.7 %)	
Postdialysis weight, kg, mean ± SD	80.4 ± 22.7	80.8 ± 22.7	77.5 ± 20.1	77.9 ± 21.4	<0.001
Vintage, months					<0.001
Median [p25, p75]	38 [20, 68]	36 [19, 63]	46 [22, 74]	43 [23, 76]	
Vascular access, n (%)					<0.001
Fistula	59,611 (64.5 %)	2118 (67.9 %)	1266 (76.3 %)	1541 (76.0 %)	
Graft	21,036 (22.7 %)	599 (19.2 %)	250 (15.1 %)	324 (16.0 %)	
Catheter	11,810 (12.8 %)	398 (12.8 %)	142 (8.6 %)	160 (7.9 %)	
Diabetes, n (%)	63,611 (68.8 %)	2173 (69.7 %)	1346 (81.1 %)	1495 (73.8 %)	<0.001
Congestive heart failure, n (%)	11,851 (12.8 %)	355 (11.4 %)	101 (6.1 %)	159 (7.8 %)	<0.001
Coronary artery disease, n (%)	7271 (7.9 %)	256 (8.2 %)	180 (10.9 %)	140 (6.9 %)	<0.001
Cerebrovascular disease, n (%)	717 (0.8 %)	33 (1.0 %)	6 (0.4 %)	10 (0.5 %)	0.03
Cancer, n (%)	1878 (2.0 %)	78 (2.5 %)	23 (1.4 %)	51 (2.5 %)	0.03
Infection, n (%)	652 (0.7 %)	23 (0.74 %)	6 (0.4 %)	10 (0.5 %)	0.25
Peripheral vascular disease, n (%)	2405 (2.0 %)	125 (4.01 %)	45 (2.7 %)	41 (2.0 %)	<0.001
Charlson comorbidity index, mean ± SD	5.49 ± 1.93	5.57 ± 1.93	5.74 ± 1.75	5.68 ± 1.84	<0.001
Ferritin, ng/mL					<0.001
Median^a^ [p25, p75]	790 [549, 1032]	841 [592, 1094]	831 [610, 1088]	694 [417, 943]	
TSAT, mean ± SD^a^	31.7 ± 14.3	32.5 ± 14.6	33.4 ± 14.8	31.9 ± 14.2	<0.001
PTH, pg/mL^a^					<0.001
Median [p25, p75]	360 [225, 563]	335 [210, 519]	315 [199, 463]	316 [201, 493]	
Kt/V, mean ± SD^a^	1.61 ± 0.32	1.68 ± 0.33	1.71 ± 0.32	1.75 ± 0.35	<0.001
Albumin, g/dL, mean ± SD^a^	3.96 ± 0.41	3.95 ± 0.41	3.98 ± 0.40	3.94 ± 0.38	<0.001

*ESA* erythropoiesis-stimulating agent, *ESRD* end-stage renal disease, *TSAT* transferrin saturation, *PTH* parathyroid hormone, *SD* standard deviation

^a^Laboratory data are the most recent value recorded on or prior to 01 January 2012

**Fig. 1:**
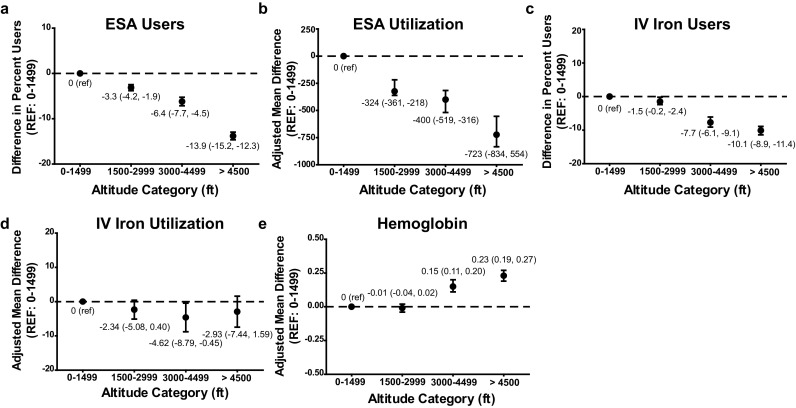
Anemia management parameters by altitude category. **a** The adjusted difference in percent users of ESA [95 % confidence interval (CI)] is presented. **b** The adjusted mean difference (95 % CI) in ESA dose among users (U/treatment) is presented. **c** The adjusted difference in percent users of IV iron (95 % CI) is presented. **d** The adjusted mean difference (95 % CI) in IV iron dose among users (mg/month) is presented. **e** The adjusted mean difference (95 % CI) in hemoglobin concentration (mg/dL) is presented. Across all panels, the 0–1499 ft category serves as the referent (ref). All analyses were adjusted for age, race, etiology, weight, vintage, access, history of diabetes, congestive heart failure, cerebrovascular disease, alcohol abuse, peripheral vascular disease, and Charlson comorbidity index

Anemia outcomes by altitude category are shown in Table 2. Compared to patients at 0–1499 ft, those in higher altitude categories (1500–2999, 3000–4499, ≥4500 ft) were less likely to receive ESA treatment. Accounting for differences in patient characteristics, altitude ≥4500 ft (vs 0–1499 ft) was independently associated with 13.9 % (95 % CI: 12.3, 15.5 %) fewer patients treated with ESA (Fig. 1a). Among ESA users, there was an incremental and monotonic association between higher altitude category and lower ESA dose. As compared to the lowest altitude category, altitude ≥4500 ft was independently associated with 723 (95 % CI: 544, 834) U/treatment lower ESA dose (Fig. 1b). Altitude≥4500 ft (vs 0–1499 ft) was also independently associated with 0.23 (95 % CI: 0.19, 0.27) g/dL higher mean Hb (Fig. 1e). A similar association was observed when ESA dose was considered per kilogram of body weight (Supplemental Fig. 1), suggesting that this finding is unlikely to be due to the fact that patients in the highest altitude category were, on average, lighter than those in the lowest category.

**Table 2:** Description of anemia outcomes by altitude category

	Altitude category (feet)			
	0–1499 (n = 92,490)	1500–2999 (n = 3118)	3000–4499 (n = 1659)	≥4500 (n = 2027)
ESA				
Utilization, U/treatment^a^				
Mean ± SD	3517 ± 3994	3063 ± 3701	2456 ± 3099	2257 ± 3191
Median [p25, p75]	2200 [1015, 4492]	1729 [769, 3977]	1523 [677, 3046]	1257 [0, 2800]
Users (%)	88.3 %	85.0 %	82.0 %	74.2 %
Adjusted difference in percent users (95 % CI)^b^	0 (ref)	−3.3 (−4.2, −1.9)	−6.4 (−7.7, −4.5)	−13.9 (−15.2, −12.3)
Dose among users				
U/treatment				
Mean ± SD	3983 ± 4027	3606 ± 3764	2995 ± 3177	3043 ± 3367
Median [p25, p75]	2454 [1354, 5077]	2200 [1185, 4485]	1862 [1185, 3575]	1833 [1015, 3758]
U/kg				
Mean ± SD	53.9 ± 58.0	48.3 ± 52.3	41.5 ± 45.6	43.1 ± 49.9
Median [p25, p75]	33.2 [17.6, 67.7]	29.8 [15.6, 60.7]	25.7 [15.3, 49.3]	25.8 [13.8, 52.9]
U/week				
Mean ± SD	11,670 ± 11,639	10,537 ± 10,857	8919 ± 9931	8990 ± 9920
Median [p25, p75]	7250 [4250,14,850]	6600 [3850, 13,225]	5500 [3300, 10,725]	5500 [3300, 11,000]
U/month				
Mean ± SD	46,680 ± 46,556	42,146 ± 43,428	35,675 ± 37,323	35,962 ± 39,681
Median [p25, p75]	29,000 [17,000, 59,400]	26,400 [15,400, 52,900]	22,000 [13,200, 42,900]	22,000 [13,200, 44,000]
U/kg/week				
Mean ± SD	157.5 ± 166.3	141.0 ± 150.2	123.4 ± 133.0	127.4 ± 147.2
Median [p25, p75]	99.2 [53.0, 199.4]	88.9 [46.9, 178.1]	77.9 [46.8, 149.3]	78.2 [41.6, 155.6]
Unadjusted modeled mean				
U/treatment (95 % CI)^b^	2599 (2585, 2613)	2278 (2206, 2351)	2002 (1932, 2074)	1871 (1795, 1949)
Unadjusted mean difference				
U/treatment (95 % CI)^b^	0 (ref)	−336 (−399, −278)	−582 (−668, −536)	−724 (−812, −647)
IV Iron				
Utilization, mg/month^a^				
Mean ± SD	162 ± 159	162 ± 150	138 ± 152	135 ± 159
Median [p25, p75]	200 [0, 200]	200 [0, 200]	150 [0, 200]	100 [0, 200]
Users (%)	73.2 %	74.6 %	65.6 %	62.8 %
Adjusted difference in percent users (95 % CI)^b^	0 (ref)	−1.5 (−2.4, −0.2)	−7.7 (−9.1, −6.1)	−10.1 (−11.4, −8.9)
Dose among users, mg/month				
Unadjusted modeled mean (95 % CI)^b^	222 (221, 222)	218 (215, 221)	211 (207, 215)	214 (210, 219)
Unadjusted mean difference (95 % CI)^b^	0 (ref)	−3.96 (−6.70, −1.22)	−10.54 (−14.66, −6.42)	−7.42 (−11.87, −2.97)
Hemoglobin, g/dL				
Mean ± SD	10.89 ± 1.15	10.91 ± 1.17	11.12 ± 1.19	11.14 ± 1.29
Median [p25, p75]	10.90 [10.20, 11.50]	10.90 [10.20, 11.50]	11.10 [10.40, 11.70]	11.10 [10.40, 11.80]
Unadjusted modeled mean (95 % CI)^b^	10.89 (10.88, 10.89)	10.91 (10.88, 10.94)	11.12 (11.07, 11.16)	11.14 (11.10, 11.19)
Unadjusted mean difference (95 % CI)^b^	0 (ref)	0.02 (0.007, 0.06)	0.23 (0.18, 0.27)	0.26 (0.21, 0.30)

All analyses were adjusted for age, race, etiology, weight, vintage, access, history of diabetes, congestive heart failure, cerebrovascular disease, alcohol abuse, peripheral vascular disease, and Charlson comorbidity index

*CI* confidence interval, *ESA* erythropoiesis-stimulating agent, *SD* standard deviation

^a^Dose among all patients, including non-users

^b^CI were generated using a bootstrapping method. Adjustments were made for covariates that were imbalanced (*P* ≥ 0.1) across groups at baseline

Patients in the highest two altitude categories were less likely to receive IV iron, while achieving higher mean Hb, compared to the lowest category. Accounting for differences in patient characteristics, altitude ≥4500 ft (vs 0–1499 ft) was independently associated with 10.1 % (95 % CI: 8.9, 11.4 %) fewer patients treated with IV iron (Fig. 1c). Among IV iron users, there was no meaningful difference in mean monthly iron dose across altitude categories, though point estimates were lower for higher altitude categories versus 0–1499 ft (Fig. 1d).

**Fig. 2:**
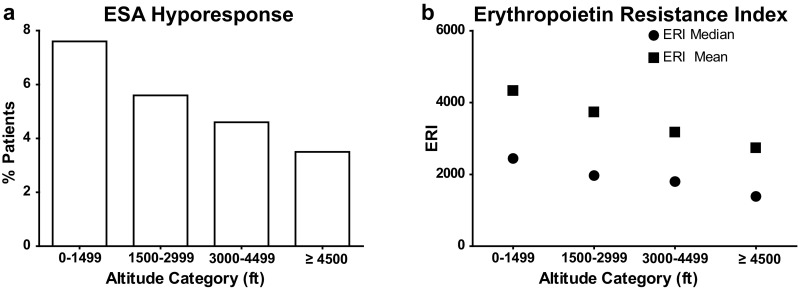
Erythropoiesis stimulating agent responsiveness metrics by altitude category. **a** The proportion of patients within each altitude category displaying ESA hyporesponse, defined as two consecutive bimonthly hemoglobin measures <10 g/dL in the setting of a concurrent averaged ESA dose >7700 U/treatment, is presented. **b** Mean (*square* symbols) and median (*round* symbols) erythropoietin resistance index (ERI) scores for patients in each altitude category are presented. ERI is calculated as the mean monthly ESA dose divided by the mean monthly hemoglobin measure

There was an incremental and monotonic association between higher altitude category and lower prevalence of ESA hyporesponse. Patients at ≥4500 ft were half as likely to be ESA-hyporesponsive as compared to patients at 0–1499 ft (3.5 % vs. 7.6 %, respectively, Fig. 2a). Correspondingly, mean and median erythropoietin resistance index (ERI) was incrementally lower with increasing altitude (Fig. 2b).

**Fig. 3:**
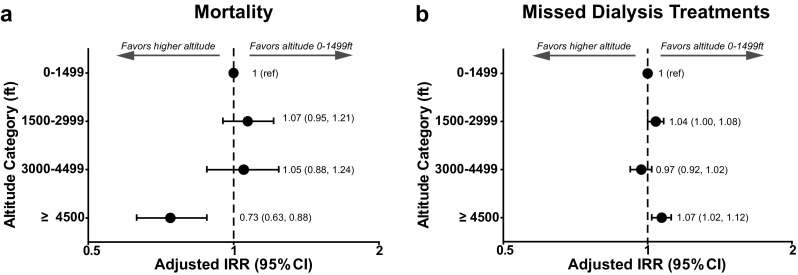
Mortality and missed dialysis treatments by altitude category. **a** The adjusted incidence rate ratio (IRR, 95 % CI) for mortality is presented for each altitude category with respect to the referent (ref) category of 0–1499 ft. **b** The IRR (95 % CI) for missed dialysis treatments is presented for each altitude category with respect to the referent category of 0–1499 ft. All analyses were adjusted for age, race, etiology, weight, vintage, access, history of diabetes, congestive heart failure, cerebrovascular disease, alcohol abuse, peripheral vascular disease, and Charlson comorbidity index

Mortality and missed dialysis treatment outcomes by altitude category are shown in Table 3. Among patients at altitude 0–1499, 1500–2999, 3000–4499 ft and ≥4500 ft, crude death rates were 10, 11, 9, and 8 deaths per 100 patient-years, respectively. Accounting for differences in patient characteristics, altitude ≥4500 ft (vs 0–1499 ft) was independently associated with a 26 % lower risk of death (adjusted IRR = 0.74; 95 % CI 0.63, 0.88; Fig. 3a). Adjusted death risk was not different for intermediate altitude categories versus 0–1499 ft.

**Table 3:** Description of mortality and missed dialysis treatments by altitude category

	Altitude category (feet)			
	0–1499	1500–2999	3000–4499	≥4500
Number of patients	92,490	3118	1659	2027
At-risk time, patient-years	80,873	2653	1481	1821
Mortality				
Number of deaths	7837	292	134	140
Crude rate^a^	10	11	9	8
Unadjusted modeled rate (95 % CI)^a,c^	9.6 (9.5, 9.9)	11 (9.8, 12.3)	9 (7.6, 10.7)	8 (6.5, 9.1)
Unadjusted IRR (95 % CI)^c^	1 (ref)	1.14 (1.01, 1.28)	0.93 (0.79, 1.11)	0.79 (0.67, 0.94)
Adjusted IRR (95 % CI)^c^	1 (ref)	1.07 (0.95, 1.21)	1.05 (0.88, 1.24)	0.74 (0.63, 0.88)
Missed dialysis treatments				
Number of missed treatments	976,200	31,707	15,321	21,924
Crude rate^b^	12.07	11.95	10.35	12.04
Unadjusted modeled rate (95 % CI)^b,c^	12.07 (11.98, 12.15)	11.94 (11.45, 12.45)	10.33 (9.78, 10.91)	12.05 (11.45, 12.69)
Unadjusted IRR (95 % CI)^c^	1 (ref)	0.99 (0.95, 1.03)	0.86 (0.81, 0.90)	1.00 (0.95, 1.05)
Adjusted IRR (95 % CI)^c^	1 (ref)	1.04 (1.00, 1.08)	0.97 (0.92, 1.02)	1.07 (1.02, 1.12)

All analyses were adjusted for age, race, etiology, weight, vintage, access, diabetes, congestive heart failure, cerebrovascular disease, alcohol abuse, peripheral vascular disease, and Charlson comorbidity index

*CI* confidence interval, *IRR* incidence rate ratio

^a^Deaths per 100 patient-years

^b^Missed treatments per patient-year

^c^CI were generated using a bootstrapping method. Adjustments were made for covariates that were imbalanced (*P* ≥ 0.1) across groups at baseline

Altitude ≥4500 ft (vs 0–1499 ft) was also independently associated with a 7 % higher rate of missed dialysis treatments (adjusted IRR = 1.07; 95 % CI 1.02, 1.12; Fig. 3b), after accounting for differences in patient characteristics. No statistical differences were detected in missed treatment rates between the other altitude groups.

## Discussion

This analysis revealed that, among contemporary ESRD patients following revised ESA dosing paradigms, residence at high altitude (≥4500 ft) was associated with improved anemia outcomes compared to residence closer to sea level (0–1499 ft). These improvements included a lower proportion of users of both ESA and iron. Iron dose among users was similar across altitude categories, likely reflecting the limited dosing options available to prescribing physicians. Although net utilization of anemia medications among patients living at higher altitudes was lower, these patients’ mean Hb was higher. Together, these trends contributed to the lower ERI scores observed at higher altitudes. Importantly, a lower incidence of ESA hyporesponse was also observed at higher altitude. This finding is clinically significant, because patients with ESA hyporesponse have worse outcomes than patients with a normal response to ESA.

The improved anemia outcomes observed in our study are consistent with previous studies conducted using patient data obtained prior to the changes in ESA dosing practices triggered by policy reform in 2011. A previous analysis found that patients residing at altitudes above 6000 ft had reduced erythropoietin (EPO) use, increased hematocrit, and decreased EPO resistance as compared to patients residing at lower elevations [11]. Similar effects were also observed when patients residing at low altitude moved to a higher altitude [10]. Although the direction of change is similar to that observed in our study, direct comparison of differences in ESA dose among altitude groups across studies is precluded by the recent changes to the US ESA labels and subsequent changes in prescribing practice.

Previous studies did not address the use of IV iron with respect to altitude. Our novel observation that the proportion of patients using IV iron is lower at higher altitudes suggests that clinically significant differences in iron utilization may exist between patients at low versus high altitude. Moreover, these results indicate that the higher Hb achieved among patients residing at higher altitude, despite lower ESA use, was not due to more aggressive treatment with IV iron.

Together, these findings are consistent with the idea that activation of the hypoxia inducible factor (HIF) family of transcription factors by the lower partial pressure of oxygen found at higher elevations leads to favorable changes in dialysis patients [21]. Potentially beneficial effects of HIF activation include mobilization of iron from storage pools, increasing synthesis of endogenous erythropoietin, and increasing the erythropoietic response to both endogenous and exogenous erythropoietin. These beneficial effects may contribute to the lower need for anemia medications among patients in our study residing at high altitude. Consistent with previous literature [12, 13], we observed a lower mortality rate among patients residing at ≥4500 ft compared to <1500 ft. This effect was observed even though our study defined altitude based on clinic zip code rather than residential zip code, used different categories of altitude, and was conducted after a marked shift in anemia treatment practices as compared to previous work. Furthermore, the present study was conducted when mortality rates were generally lower than observed during prior studies, mostly due to improvements in infection control [3]. In spite of these differences, the magnitude of the effect observed here is probably comparable to that which was reported previously.

Studies of ESA use in chronic kidney disease have shown that higher ESA doses are associated with greater mortality [22, 23]. From these prior studies, it was not clear whether the higher mortality rate was caused by the increase in Hb levels, or whether the higher mortality rate was a result of confounding (sicker patients receive more ESA, and sicker patients are also more likely to die). The results of the study presented here provide context in which to interpret these prior studies. Here, we observe a group of patients (namely those residing at high altitude) who, compared to controls (those residing at or near sea level), have similar baseline characteristics and prognostic features, and who, despite having higher Hb levels, nonetheless have better clinical outcomes, including lower mortality rates. These results suggest that it is not the increased red cell mass per se that is deleterious, but rather the conditions that lead to the need for either higher Hb or excessively high doses of ESA.

Compared to lower elevations, patients in the highest altitude category (≥ 4500 ft) had a greater missed dialysis treatment rate. Missed dialysis treatments are often due to hospitalizations (limitations in the source data precluded the ability to look at hospitalizations directly), and thus the increased missed treatment rate might suggest a higher hospitalization rate among patients living at higher altitude. However, longer travel distances and more frequent inclement weather at higher altitude clinics may also contribute to the higher missed treatment rate. Further studies, designed specifically to address this issue, are needed to clarify associations between hospitalization rates and altitude.

This study has the following limitations. First, our study was observational; associations can be measured but cause and effect are not determined. Second, despite adjustment for covariates that differed at baseline, it is possible that unknown confounding may influence study results. Third, altitude assignments were based upon clinic zip code, not residential address. Fourth, there was limited precision in point estimates at the highest altitude. Fifth, C-reactive protein values were not available, and thus inflammation status could not be determined within the study cohort.

## Conclusions

Our findings confirm that the positive association between higher altitude and patient outcomes is still evident in the context of current anemia management. Our study thus provides additional evidence to support the suggestion that this association is the result of underlying biological mechanisms, as opposed to an artifact of previous care standards. Treatments that mimic the effect of higher altitude may therefore be beneficial for patients with renal anemia.

## Electronic supplementary material

Below is the link to the electronic supplementary material.

Supplementary material 1 (DOCX 11 KB)

Supplementary material 2 (EPS 890 KB)
