# Supplementary material for: The effect of altitude on erythropoiesis-stimulating agent dose, hemoglobin level, and mortality in hemodialysis patients
Source: J Nephrol. 2016 Sep 19;30(6):821–9. doi: 10.1007/s40620-016-0350-1 (PMC5698397; doi:10.1007/s40620-016-0350-1)
Supplement: Supplementary file 1 — Supplementary material 1 (DOCX 11 KB) [file 40620_2016_350_MOESM1_ESM.docx]

**Supplemental Fig. 1 ESA dose among users by altitude category.** The mean difference (95% CI) ESA dose among users (U/kg body weight) is presented.
